# Monkeys

**Published:** 1882-04

**Authors:** 


### MONKEYS.

If the skeletons of an orang-utan and a chimpanzee be compared with that of a man, there will be found to be the most wonderful resemblances, together with a very marked diversity. Bone for bone, throughout the whole structure, will be found to agree in general form, position, and function ; the only absolute differences being that the orang has nine wrist bones, whereas man and the chimpanzee have but eight ; and the chimpanzee has thirteen pairs of ribs, whereas the orang, like man, has but twelve. With these two exceptions the differences are those of shape, proportion, and direction only, though the resulting differences in the external form and motions are very considerable. The greatest of these are that the feet of the anthropoid, or man-like apes, as well as those of all monkeys, are formed like hands, with large opposable thumbs fitted to grasp the branches of trees but unsuitable for erect walking, while the hands have weak, small thumbs, but very long and powerful fingers, forming a hook rather than a hand, adapted for climbing up trees and suspending the whole weight from horizontal branches. The almost complete identity of the skeleton, however, and the close similarity of the muscles and of all the external organs, have produced that striking and ludicrous resemblance to man which every one recognizes in these higher apes and, in a less degree, in the whole monkey tribe ; the face and features, the motions, attitudes, and gestures being often a strange caricature of humanity. Let us, then, examine a little more closely in what the resemblance consists, and how far, and to what extent, these animals really differ from us. Besides the face, which is often wonderfully human—although the absence of any protuberant nose gives it often a curiously infantile aspect—monkeys, and especially apes, resemble us most closely in the hand and arm. The hand has well-formed fingers and nails, and the skin of the palm is lined and furrowed like our own. The thumb is, however, smaller and weaker than ours, and is not so much used in taking hold of anything. The monkey's hand is, therefore, not so well adapted as that of man for a variety of purposes, and cannot be applied with such precision in holding small objects, while it is unsuitable for performing delicate operations, such as tying a knot or writing with a pen. A monkey does not take hold of a nut with its forefinger and thumb as we do, but grasps it between the fingers and the palm in a clumsy way, just as a baby does before it has acquired the proper use of its hand. Two groups of monkeys—one in Africa and one in South America—have no thumbs on their hands, and yet they do not seem to be in any respect inferior to other kinds which possess it. In most of the American monkeys the thumb bends in the same direction as the fingers, and in none is it so perfectly opposed to the fingers as our thumbs are; and all these circumstances show that the hand of the monkey is, both structurally and functionally, a very different and very inferior organ to that of man, since it is not applied to similar purposes, nor is it capable of being so applied. When we look at the feet of monkeys we find a still greater difference, for these have much larger and more opposable thumbs, and are therefore more like our hands: and this is the case with all monkeys, so that even those which have no thumbs on their hands, or have them small and weak and parallel to the fingers, have always large and well-formed thumbs on their feet. It was on account of this peculiarity that the great French naturalist Cuvier named the whole group of monkeys Quadrumana, or four-handed animals, because besides the two hands on their fore limbs, they have also two hands in place of feet on their hind limbs. Modern naturalists have given up the use of this term, because they say that the hind extremities of all monkeys are really feet, only these feet are shaped like hands; but this is a point of anatomy, or rather of nomenclature, which we need not here discuss.—Wallace, *in Contemporary Review.*

